# Supplementary material for: White matter structural bases for phase accuracy during tapping synchronization
Source: eLife. 2024 Sep 4;13:e83838. doi: 10.7554/eLife.83838 (PMC11483129; doi:10.7554/eLife.83838)
Supplement: MDAR checklist [file elife-83838-mdarchecklist1.docx]

**Materials Design Analysis Reporting (MDAR)**

**Checklist for Authors**

The [MDAR framework](https://osf.io/xfpn4/) establishes a minimum set of requirements in transparent reporting mainly applicable to studies in the life sciences.

*eLife* asks authors to **provide detailed information within their article** to facilitate the interpretation and replication of their work. Authors can also upload supporting materials to comply with relevant reporting guidelines for health-related research (see [EQUATOR Network](http://www.equator-network.org/%20)), life science research (see the [BioSharing Information Resource](http://biosharing.org/)), or animal research (see the [ARRIVE Guidelines](http://www.plosbiology.org/article/info:doi/10.1371/journal.pbio.1000412) and the [STRANGE Framework](https://doi.org/10.1038/d41586-020-01751-5); for details, see *eLife*’s [Journal Policies](https://reviewer.elifesciences.org/author-guide/journal-policies)). Where applicable, authors should refer to any relevant reporting standards materials in this form.

For all that apply, please note **where in the article** the information is provided. Please note that we also collect information about data availability and ethics in the submission form.

**Materials:**

| **Newly created materials** | **Indicate where provided: section/figure legend** | **N/A** |
| --- | --- | --- |
| The manuscript includes a dedicated "materials availability statement" providing transparent disclosure about availability of newly created materials including details on how materials can be accessed and describing any restrictions on access. | Data will be made public upon publication |  |
|  |  |  |
| **Antibodies** | **Indicate where provided: section/figure legend** | **N/A** |
| For commercial reagents, provide supplier name, catalogue number and [RRID](https://scicrunch.org/resources), if available. |  | X |
|  |  |  |
| **DNA and RNA sequences** | **Indicate where provided: section/figure legend** | **N/A** |
| Short novel DNA or RNA including primers, probes: Sequences should be included or deposited in a public repository. |  | X |
|  |  |  |
| **Cell materials** | **Indicate where provided: section/figure legend** | **N/A** |
| Cell lines: Provide species information, strain. Provide accession number in repository OR supplier name, catalog number, clone number, OR RRID. |  | X |
| Primary cultures: Provide species, strain, sex of origin, genetic modification status. |  | X |
|  |  |  |
| **Experimental animals** | **Indicate where provided: section/figure legend** | **N/A** |
| Laboratory animals or Model organisms: Provide species, strain, sex, age, genetic modification status. Provide accession number in repository OR supplier name, catalog number, clone number, OR RRID. |  | X |
| Animal observed in or captured from the field: Provide species, sex, and age where possible. |  | X |
|  |  |  |
| **Plants and microbes** | **Indicate where provided: section/figure legend** | **N/A** |
| Plants: provide species and strain, ecotype and cultivar where relevant, unique accession number if available, and source (including location for collected wild specimens). |  | X |
| Microbes: provide species and strain, unique accession number if available, and source. |  | X |
|  |  |  |
| **Human research participants** | **Indicate where provided: section/figure legend) or state if these demographics were not collected** | **N/A** |
| If collected and within the bounds of privacy constraints report on age, sex, gender and ethnicity for all study participants. | Thirty-two Mexican healthy human subjects (age= 25.37 ± 3.21 years; 19 females). Listed in Methods-Participants (page 34). |  |

**Design:**

| **Study protocol** | **Indicate where provided: section/figure legend** | **N/A** |
| --- | --- | --- |
| If the study protocol has been pre-registered, provide DOI. For clinical trials, provide the trial registration number OR cite DOI. |  | x |
|  |  |  |
| **Laboratory protocol** | **Indicate where provided: section/figure legend** | **N/A** |
| Provide DOI OR other citation details if detailed step-by-step protocols are available. |  | x |
|  |  |  |
| **Experimental study design (statistics details) *** | | |
| **For in vivo studies: State whether and how the following have been done** | **Indicate where provided: section/figure legend. If it could have been done, but was not, write “not done”** | **N/A** |
| Sample size determination | Sample size was calculated to confidently detect correlations between behavioral and diffusion metrics considering an expected r>0.5 (α=0.05; β=0.20). Methods-Participants, page 34.  (Hulley SB, Cummings SR, Browner WS, Grady D, Newman TB. Designing clinical research : an epidemiologic approach. 4th ed. Philadelphia, PA: Lippincott Williams & Wilkins; 2013. Appendix 6C, page 79.) |  |
| Randomisation | **not done** |  |
| Blinding | **not done** |  |
| Inclusion/exclusion criteria | Inclusion:   - Right-handed - Native Spanish speakers - Age range 21 – 31 - > 14 years of study   Exclusion:   - No musical training - MRI contraindications Neurological, psychiatric, or cognitive conditions.   Methods-Participants, page 34. |  |
|  |  |  |
| **Sample definition and in-laboratory replication** | **Indicate where provided: section/figure legend** | **N/A** |
| State number of times the experiment was replicated in the laboratory. |  | x |
| Define whether data describe technical or biological replicates. |  | x |
|  |  |  |
| **Ethics** | **Indicate where provided: section/submission form** | **N/A** |
| Studies involving human participants: State details of authority granting ethics approval (IRB or equivalent committee(s), provide reference number for approval. | *This study was approved by the Ethics Committee of our Institution (049H-RM).*  **Methods, page 34.** |  |
| Studies involving experimental animals: State details of authority granting ethics approval (IRB or equivalent committee(s), provide reference number for approval. |  | x |
| Studies involving specimen and field samples: State if relevant permits obtained, provide details of authority approving study; if none were required, explain why. |  | x |
|  |  |  |
| **Dual Use Research of Concern (DURC)** | **Indicate where provided: section/submission form** | **N/A** |
| If study is subject to dual use research of concern regulations, state the authority granting approval and reference number for the regulatory approval. |  | x |

**Analysis:**

| **Attrition** | **Indicate where provided: section/figure legend** | **N/A** |
| --- | --- | --- |
| Describe whether exclusion criteria were pre-established. Report if sample or data points were omitted from analysis. If yes, report if this was due to attrition or intentional exclusion and provide justification. | Exclusion criteria for sample recruitment are stated in Methods-participants (page 34). Data for one subject was discarded for Superficial White Matter analysis (figures 3, 4, and 5) due to problems in the preprocesing, specifically registration between DWI metric maps and white matter surface was suboptimal. This is stated in Superficial White Matter analysis section, page 39. |  |
|  |  |  |
| **Statistics** | **Indicate where provided: section/figure legend** | **N/A** |
| Describe statistical tests used and justify choice of tests. | Analysis of behavioral data.  Four metrics were calculated to assess the subjects’ performance during SCT (Figure 2). During the synchronization epoch, we estimated the absolute asynchronies and autocorrelation of the inter-tap interval time series (Iversen et al., 2015, Wing 2002). The constant error and temporal variability were calculated from the produced intervals during both the synchronization and continuation epochs. Absolute asynchronies were defined as the unsigned difference between stimulus onset and the tap onset. Constant error was calculated as the difference between the produced and the target interval and is a measure of timing accuracy. Temporal variability was defined as the standard deviation of the total produced intervals and is a metric of timing precision. The autocorrelation of the six inter-tap-intervals during a trial was calculated and averaged across trials. Thus, lag 1 autocorrelation is normally negative for isochrone metronomes, meaning that a longer produced interval tends to be followed by a shorter interval and vice versa, reflecting an error correction mechanism used to not lose the beat of the metronome during synchronization (Iversen et al., 2015). A repeated-measures ANOVA, with two and three factors, was carried out for the analysis of asynchronies, constant error and temporal variability.  Fixel-based analysis (FBA)  Methods-Image processing, Page 38: We analyzed individual fiber-specific properties in the presence of crossing fiber populations (‘fixels’) (Raffelt et al, 2015). For each fixel three metric were calculated: fiber density, fiber cross-section, and fiber density & cross-section (FD, FC, and FDC, respectively).  The measures of FD, FC, and FDC were correlated with the performance in the SCT (for each interval) for both conditions (visual and auditory) using a General Linear Model. Family-wise error (FWE) permutation test were carried out for multiple comparisons correction (Nichols & Holmes, 2001).  *Fixel-wise statistical results* and a specific p-value is assigned to each individual fixel (even in the presence of multiple different fixels in the same voxel). In the figure 6 and 7 we show the direct visualization of the fixel-wise statistical results by colouring each fixel according to its corrected p-value < 0.05 .  *Surface-based analysis of SWM*. Analyses were performed by fitting a general linear model at each vertex. This analysis assessed the relation between the value of diffusion metrics in each vertex (*i*) and the behavioral metrics from the SCT (absolute asynchronies, constant error, temporal variability, and lag 1 of the autocorrelation of the inter-tap-interval time series), as:  SWM *_i_* = β_0 +_ β_1_ * STC *_metric_*  Surface vertex-wise analysis was corrected for multiple comparisons using random-field theory with a threshold of family-wise error to (PFWE < 0.001). Clusters with PCluster < 0.001 were deemed significant.  Significant clusters were anatomically identified using on the Brain Atlas Based on Connectional Architecture (Brainnetome) (Fan et al., 2016). All the analyses were carried out in Surfstat (Worsley et al., 2009) for Matlab (2018; The Mathworks). |  |
|  |  |  |
| **Data availability** | **Indicate where provided: section/submission form** | **N/A** |
| For newly created and reused datasets, the manuscript includes a data availability statement that provides details for access (or notes restrictions on access). |  | X |
| When newly created datasets are publicly available, provide accession number in repository OR DOI and licensing details where available. |  | X |
| If reused data is publicly available provide accession number in repository OR DOI, OR URL, OR citation. |  | X |
|  |  |  |
| **Code availability** | **Indicate where provided: section/figure legend** | **N/A** |
| For any computer code/software/mathematical algorithms essential for replicating the main findings of the study, whether newly generated or re-used, the manuscript includes a data availability statement that provides details for access or notes restrictions. |  | X |
| Where newly generated code is publicly available, provide accession number in repository, OR DOI OR URL and licensing details where available. State any restrictions on code availability or accessibility. |  |  |
| If reused code is publicly available provide accession number in repository OR DOI OR URL, OR citation. |  | X |

**Reporting:**

The MDAR framework recommends adoption of discipline-specific guidelines, established and endorsed through community initiatives.

| **Adherence to community standards** | **Indicate where provided: section/figure legend** | **N/A** |
| --- | --- | --- |
| State if relevant guidelines (e.g., ICMJE, MIBBI, ARRIVE, STRANGE) have been followed, and whether a checklist (e.g., CONSORT, PRISMA, ARRIVE) is provided with the manuscript. |  |  |

* We provide the following guidance regarding transparent reporting and statistics; we also refer authors to [Ten common statistical mistakes to watch out for when writing or reviewing a manuscript](https://doi.org/10.7554/eLife.48175).

**Sample-size estimation**

- You should state whether an appropriate sample size was computed when the study was being designed
- You should state the statistical method of sample size computation and any required assumptions
- If no explicit power analysis was used, you should describe how you decided what sample (replicate) size (number) to use

**Replicates**

- You should report how often each experiment was performed
- You should include a definition of biological versus technical replication
- The data obtained should be provided and sufficient information should be provided to indicate the number of independent biological and/or technical replicates
- If you encountered any outliers, you should describe how these were handled
- Criteria for exclusion/inclusion of data should be clearly stated
- High-throughput sequence data should be uploaded before submission, with a private link for reviewers provided (these are available from both GEO and ArrayExpress)

**Statistical reporting**

- Statistical analysis methods should be described and justified
- Raw data should be presented in figures whenever informative to do so (typically when N per group is less than 10)
- For each experiment, you should identify the statistical tests used, exact values of N, definitions of center, methods of multiple test correction, and dispersion and precision measures (e.g., mean, median, SD, SEM, confidence intervals; and, for the major substantive results, a measure of effect size (e.g., Pearson's r, Cohen's d)
- Report exact p-values wherever possible alongside the summary statistics and 95% confidence intervals. These should be reported for all key questions and not only when the p-value is less than 0.05.

**Group allocation**

- Indicate how samples were allocated into experimental groups (in the case of clinical studies, please specify allocation to treatment method); if randomization was used, please also state if restricted randomization was applied
- Indicate if masking was used during group allocation, data collection and/or data analysis
